# Experiences of Complex Patients With Telemonitoring in a Nurse-Led Model of Care: Multimethod Feasibility Study

**DOI:** 10.2196/22118

**Published:** 2020-09-29

**Authors:** Kayleigh Gordon, Katie N Dainty, Carolyn Steele Gray, Jane DeLacy, Amika Shah, Myles Resnick, Emily Seto

**Affiliations:** 1 Dalla Lana School of Public Health University of Toronto Institute for Health Policy, Management, & Evaluation Toronto, ON Canada; 2 Center for Global eHealth Innovation Techna Institute University Health Network Toronto, ON Canada; 3 North York General Hospital North York, ON Canada; 4 Bridgepoint Collaboratory for Research and Innovation Lunenfeld-Tanenbaum Research Institute Sinai Health System Toronto, ON Canada; 5 William Osler Health System Brampton, ON Canada

**Keywords:** telemonitoring, adherence, patient experience, complex patients, normalization process theory, implementation, mobile phone

## Abstract

**Background:**

Telemonitoring (TM) interventions have been designed to support care delivery and engage patients in their care at home, but little research exists on TM of complex chronic conditions (CCCs). Given the growing prevalence of complex patients, an evaluation of multi-condition TM is needed to expand TM interventions and tailor opportunities to manage complex chronic care needs.

**Objective:**

This study aims to evaluate the feasibility and patients’ perceived usefulness of a multi-condition TM platform in a nurse-led model of care.

**Methods:**

A pragmatic, multimethod feasibility study was conducted with patients with heart failure (HF), hypertension (HTN), and/or diabetes. Patients were asked to take physiological readings at home via a smartphone-based TM app for 6 months. The recommended frequency of taking readings was dependent on the condition, and adherence data were obtained through the TM system database. Patient questionnaires were administered, and patient interviews were conducted at the end of the study. An inductive analysis was performed, and codes were then mapped to the normalization process theory and Implementation Outcomes constructs by Proctor.

**Results:**

In total, 26 participants were recruited, 17 of whom used the TM app for 6 months. Qualitative interviews were conducted with 14 patients, and 8 patients were interviewed with their informal caregiver present. Patient adherence was high, with patients with HF taking readings on average 76.6% (141/184) of the days they were asked to use the system and patients with diabetes taking readings on average 72% (19/26) of the days. The HTN adherence rate was 55% (29/52) of the days they were asked to use the system. The qualitative findings of the patient experience can be grouped into 4 main themes and 13 subthemes. The main themes were (1) making sense of the purpose of TM, (2) engaging and investing in TM, (3) implementing and adopting TM, and (4) perceived usefulness and the perceived benefits of TM in CCCs.

**Conclusions:**

Multi-condition TM in nurse-led care was found to be feasible and was perceived as useful. Patients accepted and adopted the technology by demonstrating a moderate to high level of adherence across conditions. These results demonstrate how TM can address the needs of patients with CCCs through virtual TM assessments in a nurse-led care model by supporting patient self-care and keeping patients connected to their clinical team.

## Introduction

Multimorbidity, defined as the presence of more than one chronic condition in an individual [1], has been increasing, particularly in patients with ≥4 to 5 conditions [2]. The associated risks of patients with chronic conditions include frequent hospitalizations, which can lead to increased risks of hospital-acquired infections and longer length of stay [3-5]. Previous qualitative research highlights numerous struggles patients experience in trying to manage their conditions [6], including a high treatment burden in self-managing their care, attending multiple appointments, polypharmacy, and adhering to complex care regimens [7,8]. As defined, multimorbidity does not account for common dispositions, such as frailty or aging, which may contribute to ineffective management and unanticipated outcomes. Patients with complex chronic conditions (CCCs) include both those with multimorbidity who also face complex social challenges such as socioeconomic vulnerability [9]. The layers of physical, mental, and social conditions cause extensive clinical variability in this patient population.

Several studies have found that nurse-led models enable patients to spend more time with clinicians and coordinate care under a more comprehensive and holistic approach, rather than just a single-disease focus [10-12]. Previous studies have also concluded that nurse-led models of care are feasible to deliver comprehensive chronic disease management [10,12-15] due in part to the broad scope of nursing practice, holistic approach, and interprofessional team of providers among other factors. A recent study focusing on patients with CCCs demonstrated that nurse practitioner (NP)–led care models provide sufficient quality and competency in diabetes and multimorbidity care [16]. Other studies have also found that nurse-led models of care are a potential mechanism to serve chronic populations, particularly when technology can complement patient self-care at home [15,17].

Several systematic reviews have shown that the use of telemonitoring (TM) can lead to improved health outcomes [18-23] and reduced hospital readmissions [18,24-26]. TM has also been shown to reduce all-cause mortality from heart failure (HF) [19,22,23,27], improve hemoglobin A_1c_ in patients with diabetes mellitus (DM) [28-31], improve blood pressure (BP) for patients with hypertension (HTN) [28,32,33], and reduce respiratory exacerbations in patients with chronic obstructive pulmonary disease [24]. Studies also indicate that TM can improve shared decision making [34,35] and patient experience with care [15,36]. However, several large TM trials have also reported mixed results [23,31,37-40]. It is possible that inconsistent findings are not only because of the technology itself but also because of the specific chronic conditions, combination of conditions, or lack of conditions targeted in the research. The model of care delivery in which the technology is implemented may also influence overall adherence. In particular, TM for patients with CCCs has not been widely studied in nurse-led models of care.

To address this gap, we evaluated the feasibility and perceived usefulness of the *Medly* TM system in a nurse-led care model for patients with CCCs. Feasibility is defined as the extent to which a new innovation can be successfully used or carried out within a given agency or setting [41]. The results of the implementation from the perspectives of the care team will be presented elsewhere. To the best of our knowledge, this is the first implementation of a TM system specifically targeting patients with CCCs within a nurse-led model of care. Our study was guided by the following central research question: What is the feasibility and perceived usefulness of a multi-condition TM platform in an integrated nurse-led care model?

## Methods

### Study Design Overview and Setting

A pragmatic, multimethod 6-month feasibility study was conducted for patients with CCCs in a nurse-led care clinic in Southern Ontario. The needs of patients and their families were identified in a previous qualitative study that informed the clinic’s ongoing optimization [15]. Referral criteria to the NP-led CCC clinic included patients with multimorbidity; at least one hospitalization or more than two emergency visits within the last 6 months; and a length of stay, acuity of admission, comorbidities, and emergency department visits score >5 of a total of 14 [42]. The TM feasibility study commenced approximately 12 months after the clinic launch. Patients with HF, HTN, and/or DM involved in the study were asked to take frequent physiological readings at home via *Medly*, as per Table 1. All research activities were undertaken with approval from the William Osler Office of Research Ethics (#18-0061), the University Health Network Research Ethics Board (#18-5667), and the University of Toronto Research Ethics Board (#37660).

**Table 1:** Frequency of telemonitoring readings per condition and required measures per telemonitoring algorithm.

Condition	Frequency of readings	Physiological measures
Heart failure	Daily	Blood pressure, heart rate, weight, and symptoms
Hypertension	×1 every 2 weeks	Blood pressure
Diabetes	×1 per week	Blood sugar

### Theoretical Framework

A pragmatic worldview was taken to evaluate the social construction of feasibility in multi-condition TM using the normalization process theory (NPT) by May [43-46] and the Framework of Implementation Outcomes (IOs) by Proctor et al [41]. The relationship between the 4 constructs of NPT (coherence, cognitive participation, collective action, and reflective monitoring) was evaluated using a multimethods approach in the context of the patient’s experiences of using the TM platform in the nurse-led care model. NPT was used to map the implementation process of multi-condition TM in patients with CCCs from the patient’s perspective (Textbox 1), whereas the IOs were used to determine the success or failure of the implementation using the constructs of acceptability, appropriateness, adoption, and fidelity (Textbox 2). The outcome constructs of cost, penetration, and sustainability were not specifically assessed as this study focused on the evaluation of feasibility through the patient experience and not on the model of care delivery (ie, service outcomes) or health outcomes (ie, client outcomes).

**Normalization process theory constructs and definitions.**

- Coherence

Sense making and understanding the purpose of the potential of the telemonitoring (TM) intervention

- Cognitive participation

Commitment and decision from patient (and caregivers) to commit to the work of the intervention

- Collective action

The work that patients (and caregivers) do to engage with the TM intervention

- Reflexive monitoring

Reflection and appraisal of the TM intervention

**Implementation Outcomes constructs and definitions by Proctor.**

- Appropriateness

Perceive relevance or compatibility of the innovation for a given setting or perceived fit of the innovation to address a particular issue

- Acceptability

Perception among patients that a given intervention (ie, telemonitoring [TM]) is agreeable or satisfactory

- Adoption

Initial decision or action to use an intervention (ie, TM)

- Feasibility

Extent to which a new intervention can be successfully used within a given setting

- Fidelity

Degree to which the intervention (ie, TM) was used as intended in practice

### The TM Intervention

**Figure 1:**
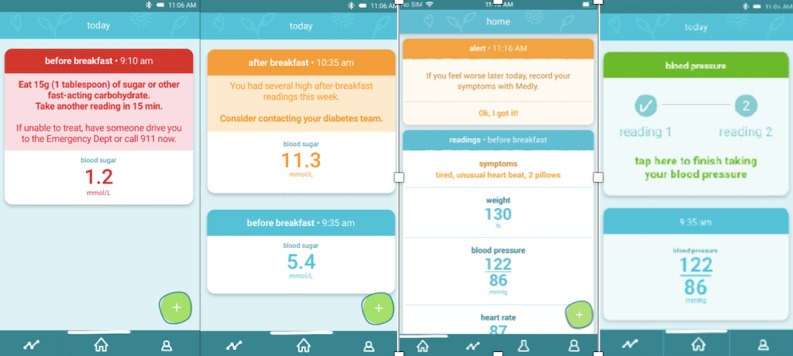
Screenshots of the Medly app for patients with a complex chronic condition.

*Medly* is a smartphone-based chronic disease TM platform that was developed by researchers at the University Health Network in Toronto, Ontario [27,47-49]. The central component of *Medly* is an app that enables patients to monitor physiological measurements (ie, BP, weight [WT], blood sugar, etc) with wireless home medical devices and to answer simple symptom questions. Readings are processed through a clinically validated algorithm embedded in the app, which is contextualized to an individual’s target range. Patients receive real-time self-care instructions, and their clinicians are alerted at the earliest signs of readings outside their individually curated normal range. Using the *Medly* app, patients were able to view graphical trends of each physiological reading over time. To assist with adherence, an automated phone call was implemented based on the required frequency of each condition’s algorithm. This call was only sent out if patients missed a reading and could be disabled on patients’ request. Participants were provided with all the necessary equipment, including a smartphone, home medical devices such as a weight scale or BP monitor, and batteries. In this case, *Medly* was designed to monitor HF, HTN, and DM (Figure 1)*.*

### Participant Sampling and Recruitment

Between May and July 2019, patients were recruited through convenience sampling during their routine visit to the nurse-led clinic. Researchers did not specifically identify patients with a specific combination of conditions, as the intent was an evaluation of feasibility. A sample of 15 to 25 participants based on specific inclusion criteria was deemed to provide sufficient data to explore the feasibility of TM in a nurse-led clinic, including reaching saturation for the patient interview data [50,51].

Patients were eligible to participate if they (1) were aged at least 18 years; (2) were able to take home measurements as intended; (3) were diagnosed with HF, HTN, or DM and one additional chronic condition; and (4) could understand English. Patients were excluded if they had visual or cognitive impairments that would prevent them from using the system or if they were already enrolled in a TM program. The NP identified which of the 3 conditions (HF, HTN, and/or DM) or a combination of conditions should be monitored for each participant. On written consent, the patient was provided with the necessary equipment and given an in-person training session on how to use the TM equipment. The NP set the specific algorithm ranges for each patient through a clinical dashboard. Patients were followed up with the* Medly* system for 6 months.

### Quantitative Data Collection and Analysis

Patient adherence to taking readings was quantitatively assessed by analyzing the server logs of the TM system. Adherence was used as a measure to indicate TM’s acceptability in this context and as an indicator of the perceived usefulness of TM. In this case, adherence was used as a pertinent implementation factor to determine feasibility [41] and was evaluated by measuring the frequency of TM readings for each condition (Table 1). For HF, as patients were instructed to take daily readings, adherence was measured as the percentage of days readings were taken over 6 months. For HTN, as per the algorithm, adherence was measured as the percentage of time patients took 2 consecutive BP readings at least once every 2 weeks. For DM, adherence was defined by taking readings at least once per week during the 6-month study.

Questionnaires were administered at the start and end of the study. Questionnaires included 3 sections: (1) the 36-Item Short Form Survey (SF-36) was used as a baseline indicator to evaluate the overall health status [52] of our participant group, (2) a series of technology questions were utilized to better understand the demographics of whom multi-condition TM is feasible within a nurse-led model, and (3) the participants’ level of comfort with technology. Determining statistical significance in a small feasibility study was not the goal of including the SF-36 tool but rather to contextualize patient health status data in this care model. A chart review was conducted to collect basic demographic information (ie, age, sex, marital status, education level, etc). Sociodemographic data and clinical histories for all participants were summarized using a descriptive analysis.

### Qualitative Data Collection and Analysis

All participants were invited to participate in a poststudy interview, each lasting between 30 and 60 min. Interviews were conducted onsite or over the telephone upon study completion. A semistructured interview guide was developed, informed by the 4 constructs of NPT (Multimedia Appendix 1) [43,44,53]. Participants were asked to describe their experiences and perspectives on using the TM system. Caregivers were not asked any specific questions during the interview but were able to participate in the discussion. All interviews were audio taped and transcribed verbatim for analysis.

An interpretative descriptive approach was used to guide the qualitative analysis [54,55], as this approach examines a clinical phenomenon with the goal of identifying themes and patterns among subjective perspectives while also accounting for variations between individuals [56]. The process of inductive thematic analysis followed the method outlined by Thorne et al [54], starting with an initial reading of each transcript. Subsequent readings included coding salient ideas and inductively deriving conceptual themes, first within and subsequently across transcripts. Detailed notes and codes were grouped according to emerging themes. The inductive process was conducted independently by 2 researchers (KG and AS) who came together to discuss emerging codes. Disagreements were discussed together and with the larger research team to reach consensus. Together, inductive codes were then compared with the implementation process constructs (NPT [43,44,46]) and the outcomes of patient experience (IOs by Proctor et al [41]) to structure the evaluation of the feasibility of this care model [41]. Saturation occurred at a point at which the codebook stabilized, a consensus was reached between the 2 researchers, and no additional codes or themes were identified in the data [57,58].

## Results

### Study Participants

In total, 26 participants identified by the team agreed to participate. Of the 26 participants, 18 were put on HF monitoring, 1 on DM, 1 on HTN, 5 on HF+DM, and 1 on HTN+DM. The remaining 8 patients were removed from the TM system for several reasons, including winter relocation (n=1), rehabilitation admission (n=1), unanticipated language barrier (n=1), inability to use a smartphone touchscreen (n=4), and death (n=1).

Table 2 presents the characteristics of the participants who completed the study. Overall, 65% (17/26) of the participants used the system for the full 6 months. The age of the participants ranged from 44 to 91 years, with a mean age of 73.8 (SD 13.3) years. All the study participants had more than 3 chronic conditions, with some participants having more than 8 conditions in total. The total number of medications ranged from 8 to 19 per participant. This feasibility study was conducted in a geographic area that had a high percentage of recent immigrants, with more than 42% of the city’s residents being born outside Canada [59]. Approximately 60% of the participants were identified as non-white. The level of education also varied widely across participants, with 35% having not completed high school and 35% having completed a college or university degree. With regard to technology experience, 11 of the 17 (65%) participants who completed the questionnaire stated that they owned a cellphone (n=4) or smartphone (n=8), whereas 3 participants did not specify which kind of device (Table 3). Only one participant had previously used a TM system, although it was not smartphone-based TM or multi-condition-based TM. Despite more than half of the participants owning a cellphone or smartphone, the comfort level across the participants was mixed. In the end, the response rate for the prestudy questionnaire was fairly high (16/17, 94%) but lower for the poststudy (10/17, 59%) questionnaires. One participant did not complete a prestudy questionnaire. An evaluation of the SF-36 data did not find any meaningful results, likely because of the low poststudy response rate.

**Table 2:** Characteristics of participants who completed the study.

Characteristics		Values, n (%)
**Sex**		
	Female	9 (53)
	Male	8 (47)
**Ethnicity**		
	White	5 (42)
	Non-White	12 (58)
**Age (years)**		
	40-49	1 (6)
	50-59	3 (18)
	60-69	3 (18)
	70-79	4 (23)
	80-89	4 (23)
	90-99	2 (12)
**Marital status**		
	Never been married	2 (12)
	Married or living with a partner	6 (35)
	Separated or divorced	2 (12)
	Widowed	6 (35)
	Missing or unknown	1 (6)
**Highest completed level of education**		
	Less than high school	6 (35)
	High school	4 (24)
	Trade or technical training after high school	1 (6)
	College or university undergraduate	6 (35)

**Table 3:** Participants’ experience with cellphones or smartphones.

Technology questions		Response, n (%)
**Own a cellphone or smartphone (n=17)**		
	Yes	11 (65)
	No	6 (35)
**If so, which kind? (n=11)**		
	Cellphone	4 (36)
	Smartphone	4 (36)
	Unknown	3 (27)
**Comfort level using a smartphone or cellphone (n=17)**		
	Not comfortable	4 (23)
	Somewhat comfortable	3 (18)
	Comfortable	3 (18)
	Very comfortable	4 (23)
	Unknown	3 (18)
**What features do you use on a smartphone? (n=17)**		
	Voice calls or text messaging	8 (47)
	Internet	6 (35)
	Video	3 (18)
	Apps or games	4 (23)
	Other	0 (0)
**If you own a smartphone, what activities do you use it for? (n=17)**		
	Email	4 (23)
	Information seeking	5 (29)
	Scheduling	2 (12)
	Information storage	6 (35)
	Recreation	5 (29)
	Other	3 (18)
	Unknown	2 (12)

### Adherence Results

Of the 17 patients who completed the study, 13 patients used the HF module, 1 patient used the HTN module, 2 patients used the HF+DM modules, and 1 patient used the HTN+DM modules. One participant (MCCP008) was initially monitoring HF and DM but was later offboarded from the DM component because of a change in health status.

Adherence to each condition was evaluated independently, irrespective of the combination of conditions. The adherence for each patient with HF is displayed in Table 4. The evaluation of adherence to HF was divided into 2 categories: the number of days patients took just the physiological readings (BP/HR/WT) and the number of days patients took all required physiological readings and completed symptom questions (full set). Although individual usage patterns varied across the participants, patients took physiological readings (BP/HR/WT), on average, over 77.2% (142/184) of days of the expected reported days. Of the patients with HF, 56% (9/16) were adherent to physiological readings over 80% of the days on TM, and 31% (5/16) were adherent over 90% of the days on TM. One patient (MCCP0017) took a full set of readings over 99% (183/184) of the days on TM. When evaluating the percentage of days that patients took all 3 physiological readings and symptom questions (ie, full set), the percentage dropped to 69.0% (127/184).

**Figure 2:**
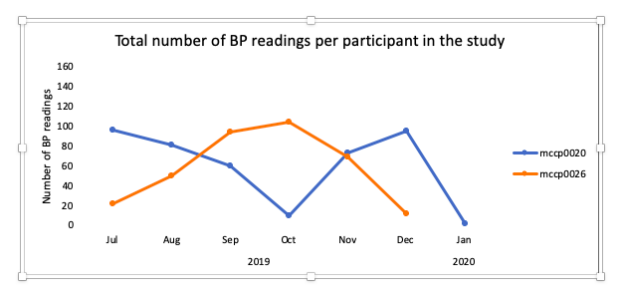
Total number of blood pressure readings on the hypertension modules. BP: blood pressure.

**Figure 3:**
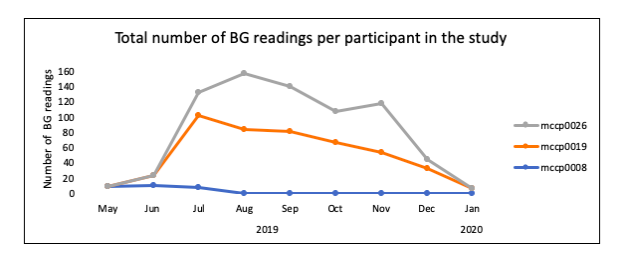
Total number of blood glucose readings on the diabetes mellitus modules. BG: blood glucose.

Overall, adherence in the HTN module was 55% (29/52) on average. HTN adherence was defined as taking at least one reading every 2 weeks. However, participants using the HTN component of the system, on average, took readings more frequently than required by the algorithm (Figure 2). DM adherence was on average 72% (19/26) defined as taking at least one reading per week (Figure 3).

**Table 4:** Heart failure adherence of physiological measures versus full set (N=184).

Study ID	Number of days taking BP^a^/HR^b^/WT^c^, n	Percentage of days taking BP/HR/WT, n (%)	Days taking full set, n (%)	Percentage delta for adherence, %
MCCP002	163	163 (88.6)	155 (84.2)	−4
MCCP005	134	134 (72.8)	122 (66.3)	−7
MCCP006	167	167 (90.8)	163 (89.6)	−2
MCCP007	147	147 (79.9)	137 (74.5)	−5
MCCP008	112	112 (60.9)	105 (57.1)	−4
MCCP009	177	177 (96.2)	177 (96.2)	0
MCCP0012	178	178 (96.7)	96 (52.2)	−45
MCCP0013	28	28 (15.2)	11 (5.9)	−9
MCCP0014	146	146 (79.3)	143 (77.7)	−2
MCCP0017	181	181 (98.4)	179 (97.3)	−1
MCCP0018	157	157 (85.3)	151 (82.1)	−3
MCCP0019	50	50 (27.2)	12 (6.5)	−21
MCCP0022	170	170 (92.4)	165 (89.7)	−3
MCCP0024	139	139 (75.5)	121 (65.8)	−10
MCCP0025	160	160 (87.9)	156 (84.8)	−2

^a^BP: blood pressure.

^b^HR: heart rate.

^c^WT: weight.

### Results From Qualitative Data

The qualitative findings of the patient experience can be represented by 4 main themes and 12 subthemes. The main themes were (1) making sense of the purpose of TM, (2) engaging and investing in TM, (3) implementing and adopting TM, and (4) perceived usefulness and perceived benefits of TM in CCCs. The themes and subthemes were mapped together with the constructs within NPT and IOs as shown in Table 5.

**Table 5:** Mapping of overarching themes and subthemes to the normalization process theory and the Implementation Outcomes framework by Proctor.

Theme and subthemes		Quotes	Normalization process theory construct	Implementation Outcomes construct by Proctor
**Making sense of the purpose of TM^a^**			Coherence	Appropriateness
		“We were keeping a log book and it wasn’t consistent but we were still trying to do that...The NP would have me email her the sugar readings. So I would have to transpose all that information into an email... they would review it when we go visit them.” (MCCP0019)		
**Engaging and investing in TM**			Cognitive participation	Acceptability
	Comparing the old ways of working to use TM	“We did] nothing. If we felt sick we’ll go to the doctor. We took blood pressure now and again, but not on a regular basis, not like this.” (MCCP0025)		
	Connected devices support individual capacity and gain investment for patient buy-in of TM practices	“I think it was we actually really liked that it was connected, because it gave me a peace of mind. At least I know, okay, if something goes wrong, there’s somebody there to call her. Someone is kind of checking on me.” (MCCP0026)		
	Accepting the technology	“Yes, it was easy. Yeah it does everything for you. You just have to turn the phone off and press the button the blood pressure cuff or step on the weight scale and it does everything.” (MCCP009)		
**Implementing and adopting TM**			Collective action	Adoption
	Adjusting routines around TM	“You get up, you put your housecoat on, you go to the bathroom, you get the scale out from under the sink.” (MCCP007)		
	Frequent clinical monitoring; reinforced routine adherence	“It’s good. It’s good to know that somebody is out here watching too not negatively. If they were watching to condemn me for what I’m doing – but they’re watching with my best interests.” (MCCP002)		
	The support of caregivers and caregiver participation	“Yeah, so it’s one hundred percent good to have help from family members.” (MCCP0019)		
**Evaluating perceived usefulness and perceived benefits of TM in CCCs^b^**			Reflexive monitoring	Feasibility and fidelity
	Improvement to ongoing self-care practices	“You don’t know whether it’s good or bad, but with Medley they give that guideline to live with – within” (MCCP002)		
	Enabling immediate action on abnormal readings and trends	“I know if the reading comes up in orange—that’s the orange. If it comes up in orange, check. If it gets worse during the course of the day check the clinic. And I know that feels good. I feel good. Yeah, I feel good, so that is very helpful, that is very, very helpful.” (MCCP002)		
	Concerns moving forward without TM	“Yeah. I like knowing someone’s keeping an eye on me. Certainly no one else will keep an eye on me now. [After I you mean?] Yes, there’s no one now...I just liked it, that’s all, knowing, they were there looking out for me.” (MCCP0024)		
	Symptom questions were not always relevant for patients with CCCs	“There were times [when it was] kind of like a grey area...She’s not always feeling great and you enter the information based on the prompts and some of those prompts alerted the nurse at the hospital and we would get a phone call. And sometimes to me they were kind of unwarranted... Was she dizzy? Well she feels dizzy a lot of times. She’s usually OK.” (MCCP0019’s caregiver)		

^a^TM: telemonitoring.

^b^CCC: complex chronic condition.

### Theme 1: Making Sense of the Purpose of TM

The first theme synthesizes participants’ accounts of how they made sense of TM, from the initial introduction to the technology to understand its purpose to enable a more structured method of physiological monitoring to connect patient information to the clinic. Participants described the process of measuring physiological readings such as BP or blood sugar at home to track their conditions for their care team. Participants were familiar with the process of keeping a logbook to track readings taken between appointments:

We were keeping a log book and it wasn’t consistent, but we were still trying to do that...

The nurse practitioner would have me email her the sugar readings. I would have to transpose all that information into an email for her... other than that everything was just in the log book and they would review it when we go visit them.

MCCP0019

One participant described how the ability to go back and look at previous readings made sense in terms of alleviating the challenge of maintaining up-to-date logs at home (ie, appropriateness). Often, manual tracking did not work because of inconsistent patient-provider communication or the inability to recall context-specific readings.

### Theme 2: Engaging and Investing in TM

By comparing the patients’ current care practices at home (ie, logs) and envisioning new work of taking digital remote readings, patients began to engage in using TM. Instead of calling in readings, by taking readings through the app, they were sent automatically to the clinic. This supported the relational work necessary to gain buy-in from patients to accept the technology and to invest time in a remote way of care.

#### Comparing the Old Ways of Working to Using TM

Before the introduction of the TM intervention, there was significant variability in the frequency of participants taking physiological readings at home routinely. Almost all participants described taking physiological readings on occasion at home, in a doctor’s office, or at a local pharmacy. One participant described their monitoring processes before beginning TM as irregular:

[We did] nothing. If we felt sick we’ll go to the doctor. We took blood pressure now and again, but not on a regular basis, not like this.

MCCP0025

One patient participant noted that she had difficulty making in-person appointments, often canceling because of the weather conditions or too many appointments. By engaging with TM, she could still receive care remotely, avoiding winter driving conditions. This contributed to increased investment in a connected TM system:

It’s hard to make appointments in this weather because you don't know what I have to come through from [city name]. You can have a big weather difference between here and there... It's hard to make every appointment with so many. I’ve had two this week already. Another at 2 and then 230 today, plus one yesterday.

MCCP0024

#### Connected Devices Support Individual Capacity and Gain Investment for Patient Buy-In of TM Practices

Having the devices connected directly to the clinic supported buy-in from patients through increased patient-clinician communication, thereby building their capacity at home (ie, saving time, providing new information to manage their care, and connecting with their personal clinicians). The ability to visualize live data and provide reassurance that someone was looking out for their health generated recurring engagement with TM and an investment to continue using the system at home:

We were keeping a log book and it wasn’tconsistent but we were still trying to do that and we’re going to have to go back to that unfortunately after this... Having this was better for us and at least for the hospitals because they could see live data right there in their systems.

MCCP0019

#### Accepting the Technology

Several participants were hesitant about the process of learning TM. Initially, several participants described the smartphone as challenging. Some participants seemed to rely more on help from family members or other informal caregivers to use TM than others:

So, the phone itself was a bit of a challenge at first. I thought “How do I go back? Where’s the back button?” All I had to do it phone you, but I figured it out... That was a little bit of a frustration...sometimes it’s better learning on your own.

MCCP0024

Overall, most patients engaged and accepted the technology over time, describing it as *easy to use*. The tasks required to complete a morning reading aligned with what their care team, particularly the nurse, was already asking them to do at home.

### Theme 3: Implementing and Adopting TM

This theme describes the process of enabling the patient’s work to implement TM at home and how these practices led to a decision to adopt the intervention seamlessly into their normal routine.

#### Adjusting Routines Around TM

The patient’s *work* of using TM frequently became a part of their everyday routine. Along with getting up, going to the bathroom, and taking their medication, taking readings was described as a normal step in this everyday process:

In the morning time when I wake up and go to the bathroom, I take my water tablet before I eat, to pass the water out. Then I test my pressure, weight, and the heart rate using Medly.

MCCP006

On the basis of the readings, patients would adjust the tasks they had done every day, for example, monitoring fluid intake or restricting salt intake.

#### Frequent Clinical Monitoring and Reinforced Routine Adherence

Participants described that they perceived someone was looking out for them (ie, nurse) and taking care of them in the virtual background (ie, nurse-led team). The registered nurse acted as a central point of contact within the care model that could address varying symptoms, cross-condition needs, and concerns. This created a mechanism that reinforced routine adherence and continued use of the TM system over time. Some participants described feeling more confident of their health knowing that someone was watching him and would reach out if something was wrong:

One thing I know is that I’m being watched. I’m being monitored. It’s nice. If something is wrong they'll know too and Medly will get in touch. It makes you more aware of what’s going on inside your body.

MCCP0025

When the clinicians would call to follow up on an abnormal reading or missed reading, participants described it as reassuring and helpful. Participants came to rely on immediate feedback not only from the team but also from the self-care messages delivered by the app:

Whenever something was wrong, the nurse would call, so it was very helpful... But like I said a couple times they just called us because of a reading they got.

MCCP0022

#### Support of Caregivers and Caregiver Participation

Participants were trained to use the TM system individually before starting the study. Although a caregiver was not required for use, 11 of 17 (65%) participants had support at home. During the poststudy interviews, it was clear that caregivers heavily participated throughout the TM process, despite whether the support was necessary:

The blood pressure taking–I have arthritis, I have rheumatism... It’s hard for me to do anything with my fingers. So it was good to have somebody to help me that way.

MCCP0014

But if I have any problems with the cell phone, I got from Medly, I will call my wife and she would play with it because I don’t have to know all this.

MCCP002

### Theme 4: Evaluating Perceived Usefulness and Perceived Benefits of TM for CCCs

In conclusion, all participants seemed to appraise the TM system as if it was already an embedded part of their daily routine in normal life. Participants described improvements in self-care knowledge, such as being able to identify when something was not right even if they felt no physical symptom differences. This enabled action on abnormal readings they may not have identified without *Medly*. Participants shared that clinicians would often quickly reach out to discuss abnormal findings. Participants described a strong desire to continue monitoring, even after the study ended. Despite high adherence rates, several technical issues presented minor challenges.

#### Improvements to Ongoing Self-Care Practices Using TM

One participant described his self-care practices before TM as just a *guessing game* (MCCP007) without clear direction or understanding how to improve this own care at home:

I was just winging it because I didn’t know anything about it at all, I weighed myself, but there was no concept behind that... now with the weight and the blood pressure together and the correlation that goes that... that aspect indicating my health [overall], it is, it was beautiful it helped me keep myself in check.

MCCP007

By taking readings daily, patients described getting to know their target range (ie, reflexive monitoring). Participants described how the device data kept them informed, improving their knowledge of their condition, and how they could keep track of their health over time (ie, feasibility).

#### Enabling Immediate Actions on Abnormal Readings and Trends

All participants referenced how *Medly* could identify when a reading or trend was not right. Many patients specifically noted using the color to identify the severity of an alert as well as the graphs within *Medly* to help identify abnormal readings:

When something is not right, your reading comes up a different color and you know something is wrong, but you can wait and retake it or you try it again on the next day.

MCCP0020

Although participants described differing preferences in how they identified abnormal readings, they described the actions they took to manage these situations:

...with Medly, when it comes up in a different color it tells you “go to see somebody if it gets worse or if you feel worse.” And what I like about it is it tells you what do to.... If your weight is not what it should be it tells your weight… I know if the reading comes up in orange, check it. If it gets worse during the day, I check with the clinic. So that is very, very helpful.

MCCP0022

Although patients described being able to more clearly identify when something was not right while using TM, many patients still relied somewhat on the nurse-led team to identify abnormalities:

They did call because they were concerned and then we did come into the clinic later that day. So maybe it was a good thing to have Medly that particular time.

MCCP0019

When a reading measures outside of the individual tailored range, participants received algorithm-based self-care messages. However, several participants found that clinicians within the model, usually the nurse, would call the patients first:

Because they’re usually pretty good. If there is a real critical [reading], they’ll try to get us right away.

MCCP0026

There were times participants even described their intention to call the nurse in the clinic; several participants noted that the nurse followed up with them before they could reach out.

#### Concerns Moving Forward Without TM

Participants expressed strong concerns about having to go back to managing their conditions at home without *Medly*, even to the point where they were willing to pay for the program out of pocket, or ask what store they could purchase the devices immediately. Participants described a sense of anxiety, knowing that no one would be monitoring their readings going forward:

Yeah. I like knowing someone’s keeping an eye on me. Certainly no one else will keep an eye on me now. [After Medly you mean?] Yes, there’s no one now...I just liked it knowing, they were there looking out for me.

MCCP0024

#### Symptom Questions Were Not Always Relevant for Patients With CCCs

Several patients described the daily symptom questions as not always relevant or specific enough to isolate subtle changes in their symptoms:

There were times [when it was] kind of like a grey area...She’s not always feeling great and we enter the information based on the prompts and some of those prompts alerted the nurse at the hospital. We would get a phone call. And sometimes, to me, they were kind of unwarranted... Was she truly dizzy? Well she feels dizzy a lot of times. She’s usually OK.

MCCP0019’s caregiver

## Discussion

### Principal Findings

This study provides a detailed evaluation of the feasibility and perceived usefulness of a multi-condition TM platform using the experiences of patients with CCCs in an integrated nurse-led model of care. Study findings revealed that patients were highly adherent to self-monitoring using multi-condition TM, irrespective of which conditions were monitored at home. A virtual connection to the nurse-led team enabled patient acceptance of this new way of tracking readings at home and engaging in their care using multi-condition TM. By choosing to adopt TM into a daily routine, patients perceived that someone was looking out for them, reinforcing routine adherence and enabling patients to evaluate abnormal readings and trends. Participants perceived TM as useful, describing improved self-care knowledge and acting on information provided by TM in tandem with their nurse-led care team. Evidence of *new normal* practices was clear (ie, high adherence rates and the patient’s detailed descriptions of perceived usefulness of TM), such that ending the study affected this new normal routine.

### Theoretical Contribution

**Figure 4:**
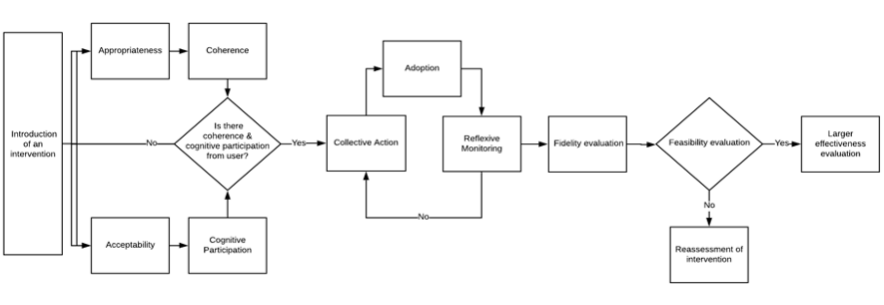
Mapping normalization process theory to Proctor’s Implementation Outcomes in a feasibility study to evaluate multi-condition in nurse-led care.

NPT and the IOs framework were used for the structural evaluation of feasibility based on the patient’s experience using TM in nurse-led care (Table 5). Using the results, a conceptual framework was developed to visualize the patient experience in light of these constructs (Figure 4).

### Simultaneous Process of Coherence and Cognitive Participation

The introduction of multi-condition TM for patients with CCCs in nurse-led care stimulated a simultaneous evaluation of acceptability (Does it make sense to the users?) and appropriateness (Does it fit?). Given these patients had frequent health system encounters that involve evaluating ongoing assessment of physiological indicators, TM of critical physiological measures aligned well with existing care plans (ie, coherence). The results indicated that patients did not always feel physical differences in their health when readings measured outside of their clinically indicated range, leading to often unanticipated condition exacerbation. Participants described the TM system as appropriate, suggesting that they understood the purpose of TM as part of their normal work, aligning with the NPT construct of cognitive participation that identifies how patients engaged with TM, accepting the technology in part as a replacement for traditional care practices. Patients accepted the idea of their *new work* in TM, in part because these devices were connected to the nurse. In many cases, an appointment was not necessary because of the virtual connections made through the TM to the clinic, creating further patient engagement and willingness to accept TM.

### A Cyclical Evaluation of Collective Action and Appraisal

Participants described an ongoing cyclical process in which the collective actions required to adopt TM resulted in a cycle of evaluation and readoption (ie, construct of reflexive monitoring) over time. In some cases, implementing TM requires embedding new routines at home or adjusting existing ones. Patients described clinicians, particularly the nurse, calling to check-in on questionable readings. Given the strong support of caregivers in our participant group, NPT might suggest that caregivers played an integral role as contributors to the relational work required in adopting TM technology (ie, collective action). The collective actions, which demonstrated engagement in the care process, reinforced patients’ reflections on the value of the technology to be positive. This led to further engagement, greater collective action, involvement of family caregivers, and overall feasibility, such that patients wanted to continue TM use after the study.

### Adherence to TM in CCCs

The results indicated an average adherence rate of 77.2% (142/184) in HF monitoring, 55% (29/52) in HTN monitoring, and 72% (19/26) in DM monitoring. Previous research has found varying adherence rates, typically between 40% and 90%, to TM in HF [47,60,61]. Another study found that the average adherence rate to BP monitoring and blood sugar monitoring was 59.7% and 50.2%, respectively [61]. In addition, other previous studies have found an initial drop in patient adherence during onboarding, followed by a steadier adherence rate over time [47,48,60]. In this case, an initial drop in adherence did not occur. A consistent overall adherence rate supports the feasibility of this multi-condition TM system. When comparing patients with HF who were adherent to physiological readings versus those who completed a full set including symptom questions, the average change in adherence was less than 10%. This suggests that the majority of patients in this cohort were adherent to both readings and symptom questions.

Although the adherence rates reported in this study are comparatively high, we anticipate that they are likely higher than reported. Similar to other *Medly* studies [47], we did not have functionality, which would enable researchers to automatically account for periods when patients were unable to take readings for legitimate reasons (eg, admitted to hospital, traveling, device replacement, etc). Patients were asked to notify the team if they were going to miss a reading. In many cases, the clinical team was notified in advance, suggesting that adherence rates are higher than reported findings. It is also possible that a subset of the incomplete readings, such as missing symptom questions, was because of the Bluetooth connectivity issues. Although patients may have taken a set of readings, it is possible that a loss in network connectivity could have reported a missed reading on that day.

### Considerations of Fidelity in Multi-Condition TM

The fidelity of the intervention was considered to contribute to the overall evaluation of TM feasibility in nurse-led care. The degree to which the intervention was delivered as intended is defined as intervention fidelity [41]. In this study, patients did not always complete the symptom questions, contributing to a difference in the adherence between a physiological reading and a full set across participants with HF and therefore lower fidelity to the original intervention. For patients with HF, adherence to completing the symptom questions was lower than completing only the physiological measures. Symptom questions may not have been completed for 3 reasons: (1) questions were not reflective of significant changes on a day-to-day basis, (2) questions were not relevant to how they felt that day (ie, determination of self-management), or (3) symptoms that are perceived as important to patients with CCCs may not be reflected in single-disease protocols. Therefore, the patient experience in the context of the combination of conditions monitored is important in evaluating the intervention’s fidelity in nurse-led care.

### Feasibility

On the basis of the patient experience, a multi-condition TM platform is feasible for patients with CCCs in an integrated nurse-led care model. Patients accepted and adopted the technology as demonstrated by a high level of adherence. Historically, the adoption and use of technology have had greater benefits in younger populations compared with older adults who may be less familiar with new technologies [62]. However, the wide age range across participants of both genders suggests the ability to use *Medly* does not appear to be associated with age or technology experience in this case. Although patients described being able to more clearly identify when something was not right while using TM, our results found that patients still relied somewhat on the clinical team to identify abnormalities. This contributed to adherence and continued use. This reliance on a clinical connection to their clinical or nurse-led team has been demonstrated in other research [15,63,64].

### Implications for Research and Future Directions

There are several implications for future multi-condition TM apps as well as scaling up existing programs that focus on populations with CCCs. Attempts to tailor specific symptom questions have already been initiated in other eHealth technologies such as the electronic patient-reported outcome tool [65]. Further research should be conducted to explore the content and frequency of symptom questions in CCCs. Given the lower level of adherence to symptom questions in this study, an evaluation may suggest subtle changes to the question content or the frequency of questions required by the algorithm. Future research could explore these adjustments within the *Medly* platform as well as other TM platforms.

More research is needed to explore the extent of caregiver support in TM as well as identify the criteria for suitable enrollment of certain patient subgroups that require caregiver support. It is possible that TM interventions for patients with CCCs could be expanded if the implications of caregiver support and the role of caregivers are more broadly understood.

Finally, given the historically high rates of health care utilization in complex populations and the need for physical distancing because of infectious diseases such as COVID-19, TM solutions that improve patient experience should be explored as viable solutions to avoid in-person appointments while continuing to monitor complex patients closely, manage care needs remotely, and mitigate unanticipated visits to the emergency department.

### Strengths and Limitations

The strengths of this study include the depth of the interview data collected as well as the rigorous approach to analysis using 2 trained qualitative coders and 2 theoretical frameworks (NPT and IOs). Interviews were candid, and diverse participants appeared to be forthcoming in their experiences using TM and perspectives on how to move forward with TM in nurse-led care. Given the nature of evaluating the initial feasibility of TM in nurse-led care for this population, the use of NPT and IOs worked well. There is an opportunity to use other well-known theoretical frameworks in future research, such as the Unified Theory of Acceptance Use of Technology 2 that undertakes a deeper dive in constructs such as price value, hedonic motivation, and effort expectancy in a program implementation.

There are several limitations to this study. First, the heterogeneous sample size was small (n=26), with only few patients using TM to monitor more than one condition. In an effort to capture the broad spectrum of CCCs, we did not attempt to randomize our sample. Only a small number of participants monitored HTN or DM exclusively. It is possible that different adherence results may have been experienced with more multi-condition TM participants. Second, as participants were conveniently sampled based on the recruitment criteria and not randomly selected, a selection bias is possible within the study sample. Third, a defective phone battery in several phones during the study generated the illusion of nonadherence, but for legitimate reasons. Owing to delayed shipping, these participants were unable to synchronize readings from the devices to the phone, likely lowering the apparent adherence data. Due to the low response rate of the SF-36, we were unable to incorporate it into our analysis. Finally, participants were only followed up for 6 months; therefore, adherence after the study period remained unknown as well as an optimal duration of TM in this population.

### Conclusions

Patients with CCCs perceived TM within a nurse-led care model to be feasible based on their experience using a multi-condition TM platform for 6 months. Overall, this study found promising adherence rates across the 3 conditions monitored by TM in this study. Patients monitoring HF demonstrated the highest rates of adherence at 77.2% (142/184) of the days in the study period. The qualitative results enabled an exploration of the feasibility of multi-condition TM, which could then be mapped to the constructs of NPT and IOs. Given the experiences of patients with CCCs, TM via multi-condition platforms in nurse-led care models should be considered to meet the growing need for virtual care interventions to support remote care of CCCs in the future.

## Appendix

Multimedia Appendix 1 Patient interview questions.

## Abbreviations

BP: blood pressure
CCC: complex chronic condition
DM: diabetes mellitus
HF: heart failure
HTN: hypertension
IOs: Implementation Outcomes
NP: nurse practitioner
NPT: normalization process theory
SF-36: 36-Item Short Form Survey
TM: telemonitoring
WT: weight
