# Addendum to the Acknowledgements: Experiences of Complex Patients with Telemonitoring in a Nurse-Led Model of Care: Multi-Method Feasibility Study

**DOI:** 10.2196/52913

**Published:** 2023-10-24

**Authors:** Kayleigh Gordon, Katie N Dainty, Carolyn Steele Gray, Jane DeLacy, Amika Shah, Myles Resnick, Emily Seto

**Affiliations:** 1 Dalla Lana School of Public Health University of Toronto Institute for Health Policy, Management, & Evaluation Toronto, ON Canada; 2 Centre for Global eHealth Innovation, Techna Institute University Health Network Toronto, ON Canada; 3 North York General Hospital North York, ON Canada; 4 Bridgepoint Collaboratory for Research and Innovation Lunenfeld-Tanenbaum Research Institute Sinai Health System Toronto, ON Canada; 5 William Osler Health System Brampton, ON Canada

In “Experiences of Complex Patients With Telemonitoring in a Nurse-Led Model of Care: Multimethod Feasibility Study” (JMIR Nursing 2020;3(1):e22118) the authors made one addition.

The following text has been added as an Acknowledgment:

This research was made possible by the funding support from a Canadian Institutes of Health Research Personalized Health Catalyst Grant (Funding Reference Number 155443).

The correction will appear in the online version of the paper on the JMIR Publications website on October 24, 2023 together with the publication of this correction notice. Because this was made after submission to PubMed, PubMed Central, and other full-text repositories, the corrected article has also been resubmitted to those repositories.

