# Supplementary material for: Experiences of Complex Patients With Telemonitoring in a Nurse-Led Model of Care: Multimethod Feasibility Study
Source: JMIR Nurs. 2020 Sep 29;3(1):e22118. doi: 10.2196/22118 (PMC8408315; doi:10.2196/22118)
Supplement: Multimedia Appendix 1 [file nursing_v3i1e22118_app1.docx]

**Multimedia Appendix**

Semi-Structured Interview Questions (Patients)

1. Tell me about your experience in the CMC so far?
   1. Prompt: Any positive or negative experiences thus far?
2. Is this clinic different from other clinics you have attended in the way it is setup?
3. How often did you visit the clinic?
   1. Did you have any issues to attend your appointments? Did you miss any appointments in the past? Why?
4. What kinds of health-related changes or decisions have been made while you have been enrolled at the CMC?
   1. Do you think you have been sufficiently involved in the decisions related to your care? How so?
   2. Prompt: Do you feel you received enough information to understand care plan?
   3. Do you feel you have received enough information to understand treatment alternatives with the health care team? If not, what information was missing?
5. Were you able to voice your concerns to the health care team?
   1. Prompt: Were you satisfied with how they responded? How so?
6. How confident did you feel about what the health care team asked for you to do?
   1. Did you have issues to remember their request?
   2. Did you have issues to understand their request?
   3. Did you have issues to accommodate their request?
7. Do you have the help or support of a caregiver (i.e. a spouse or child)?
   1. How much is this person involved in your care?
   2. Do they always come with you to your appointments?
   3. Are they aware of your condition?
   4. How do you feel about their involvement in your care?

**You have been part of the group using telemonitoring at the clinic.**

1. Tell me about your experience using the TM system?
   1. Prompt: For you, what are the main components of TM that you use?
2. How would you describe it compared to what you did before having the Medly?
   1. Prompt: Did you take readings at home before using Medly?
   2. Prompt: Does TM (Medly) fit with how you normally take care of your health? How so?
3. How involved were you in taking readings using the Medly system?
   1. Prompt: Did you have anyone help you use the system?
4. How would you describe the initial process of learning how to use Medly?
   1. Prompt: Did you feel you had enough training?
5. Based on your experiences, were there any problems with the devices at any point?
   1. Prompt: If so, could you describe what occurred?
6. Did you feel supported throughout the process?
   1. Prompt: If so, by whom?
   2. Does anyone support you at home with Medly?
7. What do you feel are the benefits of using a system like this?
8. What do you feel are the negatives?
   1. Prompt: How so?
9. Can you describe your comfort level using the TM system?
10. Can you describe if and how TM affected your interactions with the clinic staff?
11. Can you describe any challenges to using TM?
    1. Prompt: Have you adopted any strategies to overcome any of these challenges?
12. What are some ways the system could be changed or improved?
    1. Are there any features that could be added or removed?
13. Is TM something that you feel could be used in the long-term for patients for chronic conditions?
14. Is there anything that would you like to mention about your experience with TM that you don’t think we have covered?

Thank you for your time and close interview.
